# Is drug-induced toxicity a good predictor of response to neo-adjuvant chemotherapy in patients with breast cancer? -A prospective clinical study

**DOI:** 10.1186/1471-2407-4-48

**Published:** 2004-08-13

**Authors:** Chintamani, Vinay Singhal, JP Singh, Ashima Lyall, Sunita Saxena, Anju Bansal

**Affiliations:** 1 Department of Surgery, Indian Council Of Medical Research (ICMR), Institute Of Pathology Safdarjang Hospital New Delhi, India; 2 Tumor Biology Lab, Indian Council Of Medical Research (ICMR), Institute Of Pathology Safdarjang Hospital New Delhi, India; 3 Vardhman Mahavir Medical College, Safdarjang Hospital New Delhi 110023, India

**Keywords:** Neoadjuvant chemotherapy, response, drug toxicity, apoptosis

## Abstract

**Background:**

Neo-adjuvant chemotherapy is an integral part of multi-modality approach in the management of locally advanced breast cancer and it is vital to predict the response in order to tailor the regime for a patient. The common final pathway in the tumor cell death is believed to be apoptosis or programmed cell death and chemotherapeutic drugs like other DNA-damaging agents act on rapidly multiplying cells including both the tumor and the normal cells by following the same common final pathway. This could account for both the toxic effects and the response. Absence or decreased apoptosis has been found to be associated with chemo resistance. The change in expression of apoptotic markers (Bcl-2 and Bax proteins) brought about by various chemotherapeutic regimens is being used to identify drug resistance in the tumor cells. A prospective clinical study was conducted to assess whether chemotherapy induced toxic effects could serve as reliable predictors of apoptosis or response to neo-adjuvant chemotherapy in patients with locally advanced breast cancer.

**Methods:**

50 cases of locally advanced breast cancer after complete routine and metastatic work up were subjected to trucut biopsy and the tissue evaluated immunohistochemically for apoptotic markers (bcl-2/bax ratio). Three cycles of Neoadjuvant Chemotherapy using FAC regime (5-fluorouracil, adriamycin, cyclophosphamide) were given at three weekly intervals and patients assessed for clinical response as well as toxicity after each cycle. Modified radical mastectomy was performed in all patients three weeks after the last cycle and the specimen were re-evaluated for any change in the bcl-2/bax ratio. The clinical response, immunohistochemical response and the drug-induced toxicity were correlated and compared.

Descriptive studies were performed with SPSS version 10 and the significance of response was assessed using paired t-test. Significance of correlation between various variables was assessed using chi-square test and coefficient of correlation calculated by Pearson correlation coefficient.

**Results:**

There was a statistically significant correlation observed between clinical, immunohistochemical response (bcl-2/bax ratio) and the drug-induced toxicity.

**Conclusion:**

Responders also had significant toxicity while non-responders did not show significant toxicity following neoadjuvant chemotherapy. The chemotherapy-induced toxicity was observed to be a cost effective and reliable predictor of response to neo-adjuvant chemotherapy.

## Background

Neo-adjuvant chemotherapy (NACT) is an integral part of multi-modality approach in the management of locally advanced breast cancer (LABC). It is required both for the local control (to ensure microscopically free margins during surgery) and distant or systemic control [1-5]. In the past few years, considerable research has been done on the molecular aspects of breast cancer. The recognition that tumor growth rate is a product of the proliferative activity and the rate of cell death has lead to a reappraisal of traditional views of tumor response and resistance to cytotoxic Drugs [2].

Apoptosis is a closely regulated form of active cell death defined by characteristic biochemical and morphological criteria. A large number of anti-cancer agents with widely differing modes of action have been demonstrated to induce apoptosis in vitro, suggesting this as a significant final common pathway for exerting their clinical effects. Mechanisms that suppress apoptosis may be important in the development of intrinsic and acquired resistance to cytotoxic drugs [3].

It was suggested more than 20 years ago that apoptosis might account for much of the spontaneous cell loss (known from kinetic studies) to occur in many tumors and its extent often is enhanced by well-established modalities such as chemotherapy, irradiation and hormone ablation. However, during the past few years, advances in the understanding of the control of apoptosis at the molecular level has extended its potential oncologic significance far beyond the mere provision of a mechanistic explanation for tumor cell deletion. In particular, the discovery that the products of certain proto-oncogenes can regulate apoptosis has opened up exciting avenues for future research [4].

The protean effects of various neoplastic agents on synthesis of DNA, RNA, and inhibition of synthesis which may or may not lead to cell death, requires only that some critical concentrations of active drug or metabolite be present in a cell. Proliferating normal cells may therefore be subject to the same detrimental effects of chemotherapy experienced by neoplastic tissue and successful chemotherapy dictates that recuperative abilities of normal tissues are greater than those of cancer. The two tissues generally most adversely affected by antineoplastics are the hemopoetic cells of the bone marrow and the epithelium of the aero digestive tract as a result of high growth fractions and short cycling times of the cells. The ability of the cancer patients to perform normal activities and function is also recognized both as a determinant of how well the patient may respond to chemotherapy and an index of the general toxicities of the anti cancer agents.

A variety of anti-cancer drugs have been shown to induce extensive apoptosis in rapidly proliferating "normal" cell population and the tumors alike. Thus enhanced apoptosis is also likely to be responsible for many of the adverse effects observed following chemotherapy [5]. Apoptosis being the common final pathway both for tumoricidal effect and also for the toxic side effects, a significant correlation should therefore exist between the effects and the toxicity produced. Toxic effects could thus serve as reliable indicators of apoptosis

Apoptosis is a regulated phenomenon capable of being inhibited and activated. Indeed there is evidence that stimulation of some cells by trophic cytokines or increase in their levels of expression of Bcl-2 proto-ontogeny can greatly increase their resistance to the apoptosis-inducing effects of anticancer drugs. Thus Bcl-2 proto-ontogeny expression may be implicated in the development of resistance of tumors to therapeutic agents and may contribute to tumor growth and perhaps to ontogenesis by allowing the inappropriate survival of cells with DNA abnormalities [6].

Deregulated expression of the Bcl-2 protein represents the best-known example of a potent blocker of apoptosis. Over expression of Bcl-2 has now been shown to protect a wide variety of cell types from induction of apoptosis by many different anticancer agents. Several homologues of Bcl-2 protein have also been shown to act as inhibitors of apoptosis, including Bcl-Xl and others as apoptotic proteins such as Bax. In vitro data suggest that it is the relative ratios of anti-apoptotic and pro-apoptotic proteins that determine the likelihood of cells to undergo apoptosis in response to chemotherapeutic drugs [2,7]

The increasing use of pre-operative chemotherapy (PCT) in breast cancer offers an *in vivo *test bed to further confirm the clinical relevance of these observations. The clinical response or the absence of response along with the toxic effects observed could well help predict the outcome with a particular chemotherapeutic regime and facilitate planning of an alternate regime for better response [5-8].

It is vital to assess the response to NACT in order to tailor the regime for a particular patient to predict the intrinsic or acquired chemo resistance. DNA-damaging agents such as chemotherapeutic drugs can induce apoptosis and increased resistance to chemotherapeutic agents, which has been found to be associated with decreased capacity to undergo apoptosis [5-9]. Central to this are proteins that modulate apoptosis, including bcl-2 and bax products. The change in expression of apoptotic markers brought about by various chemotherapeutic regimens is being used to identify drug resistance in the tumor cells. Various other biological markers like p-glycoprotein expression have also been used to predict the response to neoadjuvant chemotherapy [9-11].

The clinical response along with complete pathological response (CPR) is still considered a surrogate marker of response against which all other predictive markers are compared. The need to have a reliable and inexpensive predictor of response in a third world scenario can not be over emphasized since majority of patients present relatively late and the resources are limited and scarce [2,3]. Since both the response and drug related toxicity due to chemotherapeutic agents are the result of the same common final pathway of apoptosis the toxicity due to chemotherapy along with clinical response may be utilized as a cost-effective and reliable indicator (predictor) of response to neo-adjuvant chemotherapy. Against this background a prospective study was contemplated with the following aims and objectives:

1. To assess the clinical and immunohistochemical response to NACT in patients with LABC.

2. To correlate immuno-histochemical (apoptotic markers i.e. Bcl-2/Bax ratio) and clinical response to the drug induced toxicity.

3. To ascertain whether the drug induced toxicity could be utilized as a reliable indicator and predictor of response to neoadjuvant chemotherapy.

## Methods

50 FNAC proven cases of locally advanced breast carcinoma according to AJCC (American Joint Committee On Cancer) classification were included in the study

A thorough clinical and ultrasonographic examination (USG) of all the patients including the opposite breast was performed to stage the disease accurately. A core biopsy using a tru-cut needle was performed for immuno-histochemical estimation of the apoptotic markers i.e. base-line Bcl-2/Bax ratio before initiating the chemotherapy. Routine and metastatic work up was done including complete blood examination (total blood count, platelet count), chest radiograph, ECG (Echocardiography when ECG had a positive finding), liver function tests, Bone Scan, USG abdomen, KFT (Kidney function tests).

Patients were subjected to three cycles of FAC regime (cyclophosphamide 600 mg/m^2^, adriamycin -50 mg/m2, 5-fluorouracil-600 mg/m2) at an interval of three weeks. Before each cycle the patient was clinically and sonologically examined for the breast tumor size, axillary lymph node status & appearance of systemic metastasis. All patients were given the same antitoxicity treatment according to a standardized unit protocol including adequate hydration and inject able antiemetics before initiating the chemotherapy.

The patients were observed for three main toxicities of the FAC regime i.e. vomiting, alopecia and leucopenia. Vomiting with minimum of four episodes on day one and two after chemotherapy was taken as significant. Total alopecia was considered significant. Leucopenia was assessed in terms of the WHO grading of hematological toxicity.

All patients were subjected to Patey's modified radical mastectomy three weeks after the last cycle and the specimen were again subjected to immuno-histochemistry to evaluate for any change in the Bcl-2/Bax ratio and for the histological tumor size, margins.

Objective clinical response was defined as >50% reduction in the tumor size after completion of three cycles of NACT, as assessed clinically, sonologically and histologically. Immunohistochemical response was taken as decrease in the Bcl-2/Bax ratio. Any increase or no change in this ratio was considered as no response.

### Immuno-histochemical methods

Biopsy specimen was preserved in buffered formalin solution. Five-micron sections were prepared from paraffin embedded blocks on poly-l-lysine coated glass slides. Sections were deparaffinized in xylene for 15 min. and hydrated in alcohol for 15 minutes. Further, incubation was done in 0.3% hydrogen peroxide in methanol solution for 45 min. The slides were washed with citrate buffer and kept in a water bath at 90–95°C for 45 min. for antigen retrieval. Sections were washed with Tris buffer saline (TBS) solution and incubated with blocking antibodies (DAKO monoclonal mouse antihuman Bcl-2 oncoprotein for Bcl-2 expression and polyclonal rabbit antihuman for Bax expression) at 37°C. Sections were washed with TBS solution. Incubation with avidin-biotin complex (ABC) was done at 37°C for one hour and washed with TBS solution. 3,3 Diaminobenzidine tetra hydrochloride solution applied for 3–5 min. Counter-staining with haemotoxylin solution done for 3–5 min. Sections were washed with distilled water, air dried and mounted using DPX mountant.

For Bax, positive controls were taken as germinal centers of the lymphoid follicles and normal breast tissue and negative control was taken as the test slide without primary antibody. For Bcl-2, positive controls were the mantle zone of lymphoid follicles and the negative controls were the test slides without primary antibody.

The pattern of positive staining for bcl-2 and bax was cytoplasmic, granular.

The primary antibodies for bcl-2 and bax were procured from DAKO.

Bax-Rabbit Anti-Human code no. A 3533.

Bcl-2 Monoclonal Mouse Anti-Human code no. M 0887.

Dilution for both was 1: 40.

The results were interpreted on the basis of two criteria:

(1) Percentage of cells showing immune bodies; <5%: score = 0, 5–25%: score = 1, 25–75%: score = 2, >75%: score = 3

(2) Intensity of staining; mild: score = 1, moderate: score = 2, intense: score = 3.

"*Since there was a strong correlation between the intensity of staining and percentage of cells showing immune bodies, the percentage of cells showing immune antibodies alone was considered for calculating the bcl-2/bax ratio*".

### Statistics

Descriptive studies were performed with SPSS version 10. The significance of response assessed using paired t-test. Significance of correlation between various variables assessed using chi-square test and coefficient of correlation was calculated by Pearson correlation coefficient.

## Results

50 cases of locally advanced breast cancer were included in this study. Mean age of the patients was 45.5 years (range: 28 to 71 years) and 53.3% patients were pre-menopausal. Size of the tumor was measured clinically as well as by ultrasound and the patients were subdivided into four groups: <5 cm(0%), 5–8 cm(56.6%), 8–10 cm(26.6%), >10 cm(16.6%). According to the axillary lymph node status the patients were divided into three groups: N0 (0%), N1 (33%), N2 (67%).

Objective clinical response was defined as more than 50% reduction in the tumor size after three cycles of neoadjuvant chemotherapy. Immuno-histochemical response was defined as decrease in the Bcl-2/Bax ratio.

Clinical response including the reduction in the tumor size and axillary lymph node status was observed in 70% of patients and was found to be statistically significant (p < 0.0001). There were no patients in the No group and 29.4%of the N1 patients were down staged to N0 while70.6% remained N1. In patients with N2 disease 7.7% were down staged to N0 status while 46.2% were downstaged to N1 status and 46.2% did not show any response. Immuno-histochemical response was observed in 60% and was also found to be statistically significant (p = 0.008). Correlation between immuno-histochemical and clinical response was also found to be statistically significant (p < 0.0001) [Table 1].

Acute vomiting was observed in 63.3% patients. 81% clinical responders had vomiting (p = 0.002) and 78% immunohistochemical responders also had vomiting which was statistically significant (p = 0.04). Alopecia was observed in 86% clinical responders (p = 0.000) and 94% immuno-histochemical responders (p = 0.000), which was also significant. Leucopoenia was observed in only 14% and 17% of clinical and immuno-histochemical responders respectively and was found to be an insignificant predictor of response in the present study. When multiple toxicities were correlated with the clinical and immuno-histochemical response, 46.7% of patients had both acute vomiting and alopecia. 67% clinical responders (p = 0.001) had both vomiting and alopecia.72% immunohistochemical responders (p = 0.001) had both vomiting and alopecia.

A significant positive correlation was observed between the presence of vomiting (r = +0.558), alopecia(r = +0.802) and response to neoadjuvant chemotherapy. A significant negative correlation was observed between the absence of side effects and poor response to neoadjuvant chemotherapy (Table 2).

## Discussion

Carcinoma of the breast is the leading cause of cancer in women all over the world and the second most common malignancy in India after carcinoma of the uterine cervix [1]. No other common epithelial cancer has been so carefully studied and so extensively characterized biologically [1,2]. In developing countries like India rate of locally advanced breast cancer at first diagnosis is estimated to be as high as 25%–30%[2,5]. The treatment of locally advanced breast carcinoma (LABC) has also evolved from primarily local modalities to treatment regimens that combine both systemic and local therapy. The realization that patients with LABC are likely to have undetectable micro metastases at diagnosis has lead to systemic treatment assuming major focus of the multi-modality approach as the studies have confirmed that surgery alone is an inadequate treatment in the management of these patients. Even aggressive surgical techniques have been observed to have a higher incidence of local recurrence in these patients [10,11]. Most importantly surgery does not change the pattern of distant failure in patients who probably have micrometastatic disease at the time of diagnosis [10-13]. Multi-modality therapy that included surgery, radiation therapy, chemotherapy, hormonal therapy has had the greatest impact on survival in patients with LABC [10-13].

### Neoadjuvant chemotherapy (NACT)

A new approach in the form of neoadjuvant chemotherapy was first reported in the 1970s and was initially utilized to convert unresectable tumors to smaller tumors making them more amenable to local control with either surgery or radiotherapy. An added advantage of this approach was the ability to assess patient's response to treatment both clinically after a defined number of courses of chemotherapy and pathologically after surgical resection. Perez and colleagues reported their results of a pilot study by the South-Eastern Cancer Study Group in 1979 that the primary tumor showed partial regression (>50%)in 65% of patients after two courses of FAC [16]. NACT has also shown benefits in the operable breast cancers by increasing the chances of breast conservation by up to 90% in some trials [10-13]. The other important advantage of NACT is that it represents an *in vivo *chemo sensitivity test for assessment of tumor response from which prognostic information can be obtained. It provides an early treatment of the micrometastatic disease, counteracting the increased growth rate possibly determined by the shrinkage of the tumor. The down staging converts an inoperable case amenable to curative resection [10-13].

### Apoptosis

Introduced by Kerr et al (1972) to describe characteristic morphological changes seen during programmed cell death [3]. It is defined as a closely regulated form of active cell death defined by characteristic biochemical and morphological criteria [3,14,15]. A wide range of anticancer drugs with widely differing modes of action have been demonstrated to induce apoptosis in vitro, suggesting this as a significant common final pathway through which they exert their clinical effect. Further more the mechanisms that suppress apoptosis may be important in the development of acquired resistance to cytotoxic drugs. Apoptosis or programmed cell death plays an important role in the regulation of tissue development, differentiation and homeostasis. It is therefore possible that deregulation of apoptosis contributes to the pathogenesis of cancer [3,13-15]. Apoptosis can be differentiated biochemically and morphologically from necrosis by the following criteria [16]:

(1) Chromophin condensation

(2) Membrane blebbing

(3) Appearance of apoptotic bodies

(4) Fragmentation of genomic DNA

Certain biochemical and genetic events have been identified that are associated with multiple cell types including mammary epithelium. These include the DNA fragmentation via end nuclease activation and cleavage of intracellular proteins, expression of bcl-2 family members, tumor suppressor gene p-53 directed events, proto-oncogene activation and activation of transmembrane receptor signaling pathways such as tumour necrosis factor [4,17-22]. Although little is known about the mechanisms, which regulate apoptosis in epithelial cells, it is conceivable that defects in apoptosis related genes are involved in the pathogenesis of human cancers. The hypothesis is supported by the fact that the tumor suppressor gene product p-53, which is frequently mutated or deleted in breast cancer, is involved in regulating apoptosis [23].

The heterogeneous nature of breast cancer has resulted in overwhelming interest in search for prognostic markers to identify patients who might benefit most from the therapeutic modalities available.

Assessment of apoptosis and individual components of apoptotic pathway are therefore relevant in determining prognosis in a particular patient [24]. DNA damaging agents such as ionizing radiations and chemotherapeutic drugs also induce apoptosis. Sakakura et al have shown an association between increased resistance to chemotherapeutic agents and decreased capacity to undergo apoptosis [25]. Central to this response are proteins that modulate apoptosis, including bcl-2 and bax gene products.

Bcl-2 is anti-apoptotic in function, whereas bax is proapoptotic and it is the interaction between the two that determines the likelihood of a tumor to undergo cytotoxic drug mediated regression. Therefore any increase in bcl-2 or decrease in bax will push the balance towards chemo resistance and an increase in bax or decrease in bcl-2 will result in increased apoptosis [26-30].

It was observed in a study conducted by Kymionis et al [15] that increase in the ratio of anti apoptotic protein bcl-2 to pro-apoptotic protein i.e. bax results in markedly enhanced resistance of tumor cell lines to the cytotoxic effects of essentially all currently available chemotherapeutic drugs.

In the present study the clinical response in terms of reduction of tumor size and immuno-histochemical response in terms of change of bcl-2/bax ratio correlated significantly with the drug induced toxicity following NACT.

### Toxicity related to chemotherapeutic agents [19-23]

The time course of various toxic manipulations depends on the drug, its dose and frequency of administration, intrinsic characteristics of the tissue of interest and any local circumstances (e.g. radiation therapy, infection, trauma). There are few general rules however like mucosal toxicities of pain, erythema, ulceration etc. occur 3–10 days after the administration of most offending drugs. Bone marrow effects can be manifestated a few days later averaging 7–14 days. The recovery of normal functioning tissues in both cases is well under way 4–5 days after the zenith of the toxic effects.

Alopecia may involve the scalp or the whole body can present within 2 weeks of the drug dose or be a progressive cumulative event. Other cumulative toxicities seen only after administration of a certain quantity of drug over some length of time include cardiomyopathy from anthracyclins.

### Cyclophosphamide

An alkylating agent belonging to the nitrogen mustard subgroup. It is inactive as such produces few acute effects and is not locally damaging. It is transformed in to active metabolites like aldophosphamide and phospharimide mustard in the liver, which then produces the wide variety of antitumour action. The prominent side effects are alopecia and cystitis, which are caused by another metabolite acrolein.

### 5-Fluorouracil

It is a pyrimidine antagonist and is converted in the body to the corresponding nucleotide, 5-fluro-2-deoxy-uridine monophosphate, which inhibits thymidine synthetase and blocks the conversion of deoxyuridilic acid to deoxythymidilic acid. Selective failure of DNA synthesis occurs due to non-availability of thymidylate. Flourouracil may itself get incorporated in to nucleic acids and this may contribute to its toxicity. Even resting cells are affected, though multiplying cells are more susceptible like the cells in the gastrointestinal tract (GIT) and bone marrow.

### Doxorubicin

It is an anti tumor antibiotic and is capable of causing breaks in the DNA strands by activating topoisomerase II and generating quinone type free radicals. They have mutagenic potential. Maximum action is exerted at S phase, but toxicity is usually exerted in G2 phase. Cardiotoxicity is a unique side effect. Rapidly multiplying cells are more susceptible therefore it also acts on cells of GIT, bone marrow in addition to the tumor cells.

Leucopenia has not been observed to be a frequently encountered chemotherapy induced toxicity using commonly used regimen in most of the studies [19-24]. This was observed in the present study also.

## Conclusions

The rapidly proliferating normal and tumor cells are more susceptible to the action of chemotherapeutic agents, which could explain the significant correlation observed between the effects and the toxicity in the present study. There was a strong correlation observed between the immunohistochemical response (bcl-2/bax ratio), clinical response and drug toxicity. This indirectly indicates a correlation between chemotherapy induced apoptosis and the toxicity and therefore like apoptotic markers, chemotherapy induced toxic effects along with objective clinical response could serve as reliable and cost-effective indicators or predictors of response to NACT in patients with LABC. While many biological markers are in use and many are under trial to tailor the chemotherapy for a particular patient, most of these markers including apoptotic markers or p-glycoprotein etc. are not very frequently available and are expensive for a third world cancer set up. Thus the chemotherapy-induced toxicity along with clinical response may be utilized as a cost effective and reliable predictor of response to NACT in patients with LABC. This would also serve as an intermediate end point in determining drug sensitivity for adjuvant treatment, especially when adjuvant therapy is planned with the same regimen as induction chemotherapy. This can also help in planning an alternative regime in non-responders.

## Competing interests

None declared.

## Authors' contribution

• **CM**, the principal and the corresponding author was the Supervisor and the Chief surgeon who performed and standardized surgery on the patients and designed the study.

• **VS **participated in the designing of the study, performed the statistical analysis and was the first surgical assistant and Senior Postgraduate in charge of the cases in the study.

• **JP**, Postgraduate surgical resident was the second assistant in charge of the cases and participated in the sequence alignment.

• **AL, **Resident surgery participated in the data processing and statistical analysis.

• **SS **was in charge of the molecular genetic studies at the Tumor biology lab ICMR.

• **AB **participated in the genetic studies and data processing.

All authors read and approved the final manuscript.

## Pre-publication history

The pre-publication history for this paper can be accessed here:



## Figures and Tables

**Figure 1:**
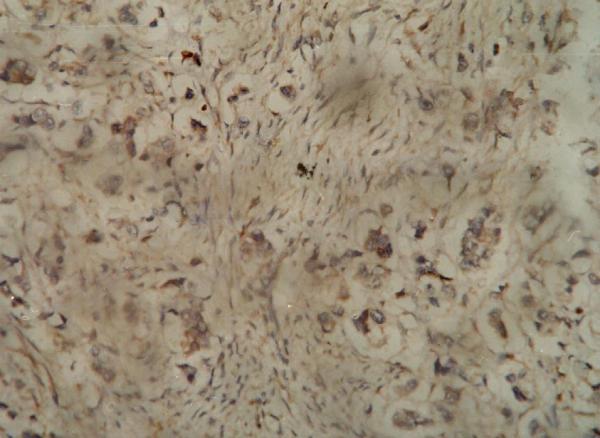
Immuno-histochemistry showing positive (moderate) staining of the tumor cells for Bax antibody.

**Figure 2:**
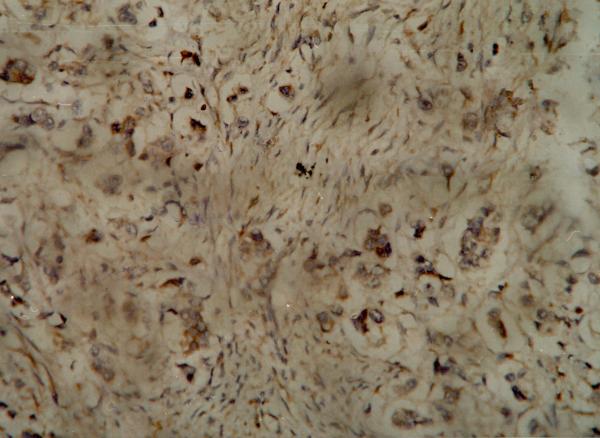
Immuno-histochemistry showing positive (intense) staining of the tumor cells for Bax antibody.

**Figure 3:**
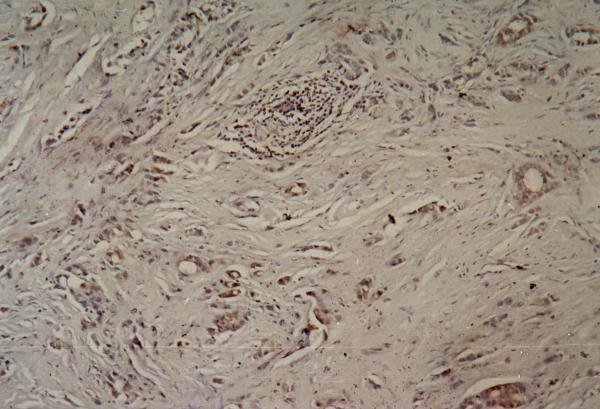
Immunohistochemistry showing positive (moderate) cytoplasmic staining of the tumor cells for the Bcl-2 antibody.

**Figure 4:**
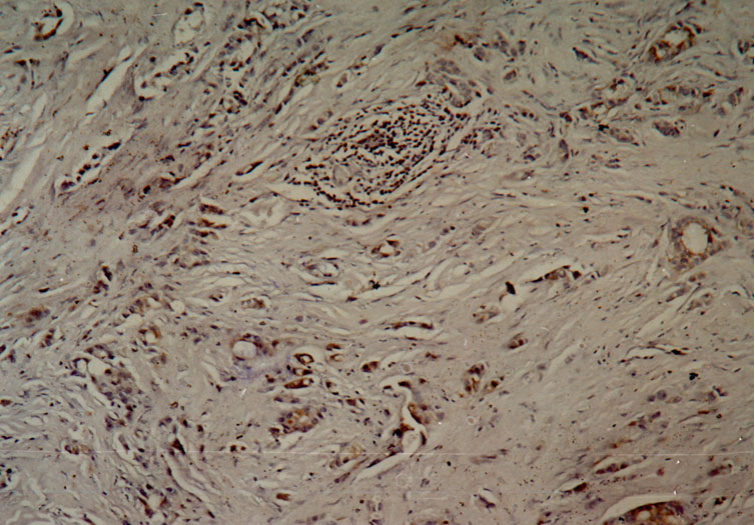
Immuno-histochemistry showing positive (intense) cytoplasmic staining of the tumor cells for Bcl-2 antibody.

**Table 1:** n = 50

		Clinical response	Clinical no response	Immuno-histochemical response	Immuno-histochemical no response
Mean age	45.5 years				
Range	28–71 yrs				
Menopausal status					
Pre-menopausal	53.3%	62.5%	37.5%	56.2%	43.8%
Post-menopausal	46.7%	71.4%	28.6%	64.2%	35.8%
Tumor size					
<5 cm	0%	-	-	-	-
5–8 cm	56.6%	68.7%	31.3%	60%	40%
8–10 cm	26.6%	75%	25%	60%	40%
>10 cm	16.6%	66.6%	33.3%	60%	40%
Axillary lymph nodes					
N0	0%	-	-	-	-
N1	33%	29.4%(toN0)	70.6%	60%	40%
N2	67%	7.7%(N0), 46.2%(N1)	46.2%	60%	40%
Total		70% (p < 0.0001)	30%	60%(p = 0.008)	40%

**Table 2:** n = 50

	Total N = 50	Clinical responders N = 35	Clinical non-responders N = 15	Immuno-histochemical responders N = 30	Immuno-histochemical non-responders N = 20
Acute vomiting	63.3%	81%(p = 0.002)	22.2%	78%(p = 0.04)	41.7%
Alopecia	60%	86%(p = 0.000)	0%	94%(p = 0.000)	8.3%
Leucopenia	10%	14%(NS)	0%	17%(NS)	0%
Alopecia+Acute vomiting	46.7%	67%(p = 0.001)	0%	72%(p = 0.001)	8.3%
